# Circulating Secretoglobin Family 1A Member 1 (SCGB1A1) Levels as a Marker of Biomass Smoke Induced Chronic Obstructive Pulmonary Disease

**DOI:** 10.3390/toxics9090208

**Published:** 2021-08-31

**Authors:** Vivek Vardhan Veerapaneni, Swapna Upadhyay, Tania A. Thimraj, Jayaraj Biligere Siddaiah, Chaya Sindaghatta Krishnarao, Komarla Sundararaja Lokesh, Rajesh Thimmulappa, Lena Palmberg, Koustav Ganguly, Mahesh Padukudru Anand

**Affiliations:** 1 Department of Pulmonary Medicine, JSS Medical College and Hospital, JSS Academy of Higher Education and Research, Mysuru 570015, India; vivekswasa@gmail.com (V.V.V.); drjayarajbs@yahoo.com (J.B.S.); chaya.sindaghatta@gmail.com (C.S.K.); kslokesh@gmail.com (K.S.L.); 2 Unit of Integrative Toxicology, Institute of Environmental Medicine (IMM), Karolinska Institutet, 17177 Stockholm, Sweden; swapna.upadhyay@ki.se (S.U.); tat2151@cumc.columbia.edu (T.A.T.); lena.palmberg@ki.se (L.P.); 3 Department of Biochemistry, JSS Medical College and Hospital, JSS Academy of Higher Education and Research, Mysuru 570015, India; kumar_rt@yahoo.com

**Keywords:** COPD, asthma, lung, pollution, CC10, CC16, CCSP, SCGB1A1, biomass, tobacco

## Abstract

Secretoglobin family 1A member 1 (SCGB1A1) alternatively known as club cell protein 16 is a protective pneumo-protein. Decreased serum levels of SCGB1A1 have been associated with tobacco smoke induced chronic obstructive pulmonary disease (TS-COPD). Exposure to biomass smoke (BMS) is an important COPD risk factor among women in low and lower-middle income countries. Therefore, in a cross-sectional study (*n* = 50/group; total 200 subjects) we assessed serum SCGB1A1 levels in BMS-COPD subjects (11 male, 39 female) compared to TS-COPD (all male) along with TS-CONTROL (asymptomatic smokers, all male) and healthy controls (29 male, 21 female) in an Indian population. Normal and chronic bronchitis like bronchial mucosa models developed at the air–liquid interface using human primary bronchial epithelial cells (3 donors, and three replicates per donor) were exposed to cigarette smoke condensate (CSC; 0.25, 0.5, and 1%) to assess SCGB1A1 transcript expression and protein secretion. Significantly (*p* < 0.0001) decreased serum SCGB1A1 concentrations (median, interquartile range, ng/mL) were detected in both BMS-COPD (1.6; 1.3–2.4) and TS-COPD (1.8; 1.4–2.5) subjects compared to TS-CONTROL (3.3; 2.9–3.5) and healthy controls (5.1; 4.5–7.2). The levels of SCGB1A1 were positively correlated (*r* = 0.7–0.8; *p* < 0.0001) with forced expiratory volume in 1 s, forced vital capacity, their ratios, and exercise capacity. The findings are also consistent within the BMS-COPD sub-group as well. Significantly (*p* < 0.03) decreased SCGB1A1 concentrations were detected with severity of COPD, dyspnea, quality of life, and mortality indicators. In vitro studies demonstrated significantly (*p* < 0.05) decreased SCGB1A1 transcript and/or protein levels following CSC exposure. Circulating SCGB1A1 levels may therefore also be considered as a potent marker of BMS-COPD and warrant studies in larger independent cohorts.

## 1. Introduction

Secretoglobin family 1A member 1 (SCGB1A1), alternatively known as club cell protein 16 or 10 (CC16 or CC10), is a protective pneumo-protein present in normal airway secretions [1]. The expression of SCGB1A1 is restricted to the airway club cells (formerly also known as Clara cells) and has several functions including anti-inflammation, inhibition of phospholipase A2, and the sequestering of hydrophobic ligands [1]. Reduced serum, sputum, and bronchoalveolar lavage fluid SCGB1A1 levels have been associated with increased tobacco smoke induced chronic obstructive pulmonary disease (TS-COPD) severity and accelerated lung function decline [2]. COPD is one of the most common cause of global mortality and morbidity accounting for three million deaths in 2015 [3]. The global burden of disease study listed COPD as the 8th most common causes of disability in the world [3]. Nearly 3 billion people globally, mainly in low and lower-middle income countries, are exposed to biomass smoke (BMS) during cooking and heating in contrast to about 1 billion tobacco smokers [4]. Women and children are disproportionately affected due to BMS exposure during prolonged cooking hours (4–6 h/day) [4]. Biomass smoke induced COPD (BMS-COPD) remains an under-researched public health topic. The prevalence of COPD in India is 4.2%, but due to the large population, the number of COPD cases in 2016 exceeded 55 million [5]. Chronic respiratory diseases mainly led by COPD are the second most common cause of death in India [6]. Even though the men versus women smoker’s ratio in India is 9:1, the corresponding ratio of COPD patients is 1.5:1 [7,8]. This is mainly driven by the high incidence of BMS-COPD among women, particularly in rural India. However, biomarker discovery studies on BMS-COPD are rare.

Therefore, in this study, we assessed serum SCGB1A1 concentration in a South Indian population consisting of BMS-COPD, TS-COPD, asymptomatic smokers (TS-CONTROL), and healthy control subjects. Due to socio-cultural reasons women in rural India are generally non-smokers and men go out for work during cooking hours. Thus, in the study cohort BMS exposed subjects are mainly women whereas tobacco smokers are only men. Further, to determine the plausible response of SCGB1A1 transcript and protein secretion following tobacco smoke exposure locally, we performed exposure studies using physiologically relevant normal-and chronic bronchitis-like bronchial mucosa models developed using human primary bronchial epithelial cells cultured at the air–liquid interface.

## 2. Material and Methods

### 2.1. Study Settings, Design and Subjects

**Figure 1:**
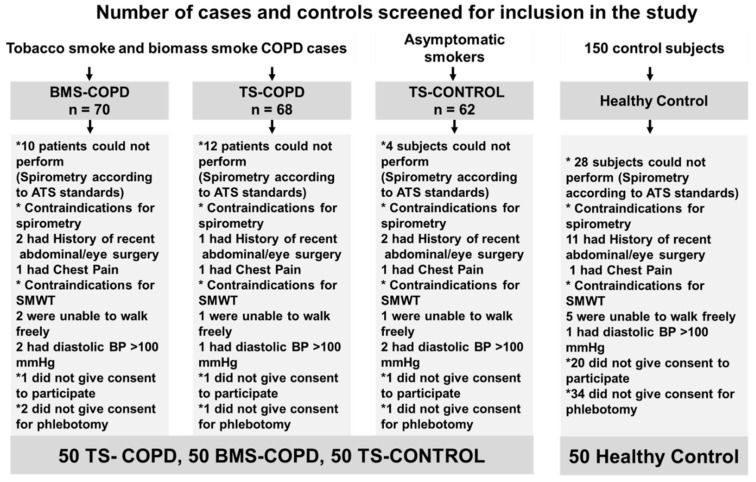
Inclusion and exclusion criteria of the study to select chronic obstructive pulmonary disease (COPD; both TS-COPD and BMS-COPD) subjects, asymptomatic smokers (TS-CONTROL), and healthy control subjects. ATS: American Thoracic Society, BMS-COPD: biomass smoke induced COPD, BP: blood pressure, SMWT: six-minute walk test, TS-COPD: tobacco smoke induced COPD.

A total of 200 subjects satisfying the inclusion criteria (Figure 1, Table 1), were recruited from a tertiary care university teaching hospital and from the community (Mysuru district, Karnataka, India). The study group consisted of 50 subjects with TS-COPD and 50 with BMS-COPD recruited from the hospital. Asymptomatic smokers (TS-CONTROL; *n* = 50) and 50 healthy controls were recruited from the community from the burden of obstructive lung disease (BOLD) cohort (Mysuru) and the Mysuru stUdies of Determinants of Health in Rural Adults (MUDHRA) after obtaining informed consent. All procedures of the human study (Indian cohort) were approved by the Institutional Ethical Committee of JSS Medical College according to the guidelines of Indian Medical Research Council (JSSMC/PG/2512/2017-18). The inclusion criteria were adults aged more than 40 years who gave informed consent, diagnosis of COPD according to Global Initiative for Chronic Obstructive Lung Disease (GOLD) guidelines, cases had to have exposure to one of the two risk factors [i.e., tobacco smoking (TS-COPD) or using biomass fuel for cooking purposes (BMS-COPD)]. Subjects with similar exposure to tobacco smoking but with normal spirometry were also recruited in the study (TS-CONTROL). Healthy controls without any co-morbidities and no exposures were selected from the general population from the BOLD cohort from Mysuru. The exclusion criteria were age less than 40 years, subjects having contraindication for spirometry as determined by spirometry safety questionnaire, history of abdominal/chest/eye surgery, myocardial infarction in past 3 months, chest/abdominal pain, and respiratory infections in past 3 weeks. Other exclusion criteria were subjects who were seriously ill, bed ridden, known history of any malignancy, subjects with active pulmonary tuberculosis, subjects with contraindications or high risk to perform six-minute walk test [9] such as a medical history of myocardial infarction or unstable angina or cardiac illness, or history of chest pain on exertion, resting pulse rate of more than 120/min, resting systolic blood pressure more than 180 mm Hg, diastolic blood pressure more than 100 mm Hg, subjects who are not able to perform six-minute walk test like physical disability, loss of limb, wounds, fractures or unable to walk, and subjects not giving written consent.

Subjects were interviewed with questionnaires which included basic demographic information, detailed medical treatment, occupation, tobacco smoking and biomass smoke exposure, quality of life questionnaires [COPD-specific version of the St. George’s Respiratory Questionnaire (SGRQ-C)] [10] and COPD assessment test (CAT) questionnaires [11]. Participants underwent six-minute walk test and spirometry (pre-and post-bronchodilator test after 400 µg of salbutamol) according to American Thoracic Society (ATS) guidelines [12]. Diagnosis of COPD was performed according to the GOLD criteria with a post bronchodilator FEV_1_/FVC <0.7. The severity of COPD was assessed according to GOLD criteria; stage II (FEV_1_ 50–80% predicted), stage III (FEV_1_ 30–50% predicted), and stage IV (FEV_1_ <30% predicted). Spirometry (Easy one PC, NDD, Medizintechnik AG, Zurich, Switzerland) was done by trained staff in the community and the predicted values for FEV_1_ and FVC were obtained using corrected Knudson’s predicted equation for Asian population (Knudson 1983) [13]. Both TS-COPD and asymptomatic smokers (TS-CONTROL) had to smoke at least ≥10 pack-years. Biomass exposure index (BMEI) was calculated by multiplying the number of hours of cooking with biomass fuel per day and the number of years of cooking with biomass fuel in their lifetime [14]. Healthy control subjects had never smoked in their lifetime and never used biomass fuel for cooking, aged 40 –75 years, and had normal lung function with a post-bronchodilator test (FEV_1_ >80% predicted and FEV_1_/FVC >0.7). BODE index [Body mass index (BMI, B), airflow obstruction (O), dyspnea (D; measured with the use of the modified Medical Research Council/mMRC dyspnea scale), and exercise tolerance (E)] were calculated as per recommendations [15].

Whole blood (3 mL) was collected by a trained technician into the vacutainer tubes and serum prepared by centrifugation at 1500× *g* for 15 min and stored at −80 °C until analysis as per standard procedure. Serum SCGB1A1 concentration was measured using a commercially available enzyme linked immuno sorbent assay (ELISA) kit (RD191022200; Biovendor-Research and Diagnostic products, Brno, Czech Republic) according to the manufacturer’s instructions.

### 2.2. Exposure of Normal-and Chronic Bronchitis-like Bronchial Mucosa Models to Cigarette Smoke Condensate

Human primary bronchial epithelial cells (PBEC) were cultured at the air–liquid-interface (ALI) to develop physiologically relevant normal (PBEC-ALI) and chronic bronchitis-like bronchial mucosa models (PBEC-ALI/CB). The PBEC-ALI models were developed using PBEC from three donors (N = 3) and three technical replicates per donor (*n* = 3). The *chronic bronchitis-like* bronchial mucosa model was developed by adding 1 ng/mL interleukin 13 (IL13) in the basal medium of each insert (PBEC-ALI/CB; N = 3, *n* = 3). The detailed protocol and details of cellular differentiation (club cells, goblet cells, basal cells, ciliated cells, etc.) of the PBEC-ALI and PBEC-ALI/CB model have been described previously [16]. The cells used in this study are well characterized and have been used in connection with several other projects [16,17,18]. The PBEC were harvested from healthy bronchial tissues obtained from donors in connection with lobectomy following their written and informed consent. All procedures performed for the in vitro study were in accordance with the approval of the Swedish Ethical Review Authority (Institutional ethic committee reference number 99-357).

Cigarette smoke condensate (CSC; 4%) was purchased from Murty Pharmaceuticals, Inc (Kentucky, KY, USA). The CSC was prepared by smoking University of Kentucky’s 3R4F standard research cigarettes on an FTC Smoke Machine. We exposed each insert to 0.25%, 0.5%, and 1% CSC by adding CSC solution (dissolved in a small volume cell culture media; total volume: 80 µL) on the apical surface of PBEC-ALI and PBEC-ALI/CB models. Following 24 h of incubation, the transcript expression of *SCGB1A1* was assessed using quantitative real time polymerase chain reaction (qRT-PCR) as explained previously [18]. Sham exposed samples (80 µL cell culture media) served as control and actin beta (*ACTB*) was used as the reference gene. The concentration of secreted SCGB1A1 protein in the basal media of PBEC-ALI and PBEC-ALI/CB was measured by ELISA using a commercially available kit (R&D systems; Minneapolis, MN, USA, DY4218) according to the manufacturer’s instruction. Cytotoxicity of the CSC doses on PBEC-ALI and PBEC-ALI/CB models was assessed by lactate dehydrogenase assay (LDH; Thermo Fisher Scientific, Rockford, IL, USA) in the basal medium 24 h post-exposure according to manufacturer’s instruction.

### 2.3. Statistics

Serum SCGB1A1 concentrations are expressed as median and interquartile range (25th–75th percentiles). Non-parametric Kruskal–Wallis followed by Mann–Whitney *U* test (two tailed), when appropriate, was performed to determine the statistical significance for the difference of SCGB1A1 concentrations between TS-COPD, TS-CONTROL, BMS-COPD, healthy controls and between male and female subjects within groups (when applicable). Two tailed Spearman’s rank test was used to perform correlation analysis between SCGB1A1 concentration with age, lung function parameters, six-minute walk distance (SMWD), and exposure indices (pack-year and BMEI). The qRT-PCR and ELISA results are also expressed as median and interquartile ranges (25th–75th percentiles) and normalized to respective sham followed by non-parametric statistical analysis. All statistics were performed to their own sham. Within each group (PBEC-ALI or PBEC-ALI/CB models), the comparisons between sham and different CSC exposure concentrations were assessed by Friedman test followed by Wilcoxon signed-rank *t*-test as a post hoc test. A *p*-value <0.05 was considered as significant. All the data and statistical analysis were performed using GraphPad Prism software (Version: 5; LaJolla, CA, USA) and STATISTICA9 software (StatSoft, Inc., Uppsala, Sweden). Detailed analysis is provided in Supplementary Tables S1–S5. This includes gender-based stratification analysis (Supplementary Table S1), age-based stratification analysis (Supplementary Table S2), correlation analysis with pre-and post-bronchodilator test of lung function (Supplementary Table S3), and disease severity-based stratification analysis in BMS-COPD subjects (Supplementary Table S4) with serum SCGB1A1 concentrations. Supplementary Table S5 includes detailed analysis of SCGB1A1 transcript and secreted protein levels following in vitro CSC exposure studies.

## 3. Results

### 3.1. Study Cohort

The cohort demography and characteristics are summarized in Table 1. A total of 350 subjects were screened to recruit the required 200 subjects for this study, which included 50 subjects/group representing TS-COPD (all male), asymptomatic smokers (TS-CONTROL; all male), BMS-COPD (male: 11; female: 39, all non-smokers), and healthy control subjects (male: 29; female 21, all non-smokers and no cooking related BMS exposure). The inclusion criteria used for subject recruitment are shown as a flow diagram (Figure 1). Briefly, there were 140 males and 60 females recruited for this study. The two important risk factors for COPD evaluated were tobacco smoking and biomass smoke exposure. All the tobacco smoke related COPD were males, and the majority of biomass exposure related COPD were females (78%). The males with BMS-COPD (22%) were cooks and factory workers exposed to biomass smoke for many hours every day. Exposure to tobacco smoke and biomass smoke are represented as pack-year and biomass exposure index (BMEI) [14] respectively.

### 3.2. SCGB1A1 Levels in BMS-COPD and TS-COPD Subjects

**Figure 2:**
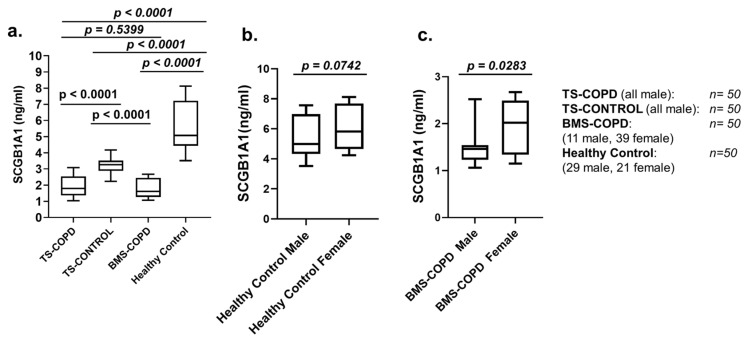
Serum concentrations of secretoglobin family 1A member 1 (SCGB1A1) among: (**a**) tobacco smokers with chronic obstructive pulmonary disease (TS-COPD), tobacco smokers without COPD (TS-CONTROL; asymptomatic smokers), biomass smoke induced COPD (BMS-COPD), and healthy control subjects (*n* = 50 subjects/group). (**b**) SCGB1A1 levels were similar among male and female healthy control subjects. (**c**) Slightly higher SCGB1A1 levels were detected among female BMS-COPD subjects compared to male BMS-COPD subjects. Data are presented as median (25th–75th percentile) and range. Statistical analysis was performed using the non-parametric Kruskal–Wallis followed by Mann–Whitney U tests (two tailed), when appropriate. *p* < 0.05 was considered as statistically significant.

Significantly decreased SCGB1A1 levels (ng/mL; interquartile range) were detected in BMS-COPD (1.61; 1.29–2.44) and TS-COPD (1.80; 1.38–2.51) subjects compared to both TS-CONTROL (3.27; 2.90–3.51) and healthy controls (5.07; 4.47–7.20) (Figure 2a). The levels of SCGB1A1 of BMS-COPD and TS-COPD subjects were comparable (Figure 2a). The concentrations of SCGB1A1 of male healthy control and female healthy controls were similar with no statistical difference (Figure 2b). The levels of SCGB1A1 in BMS-COPD-female were slightly higher (*p* = 0.03) than that of BMS-COPD-male (Figure 2c).

### 3.3. SCGB1A1 Levels in BMS-COPD Subjects Classified According to Disease Severity

**Figure 3:**
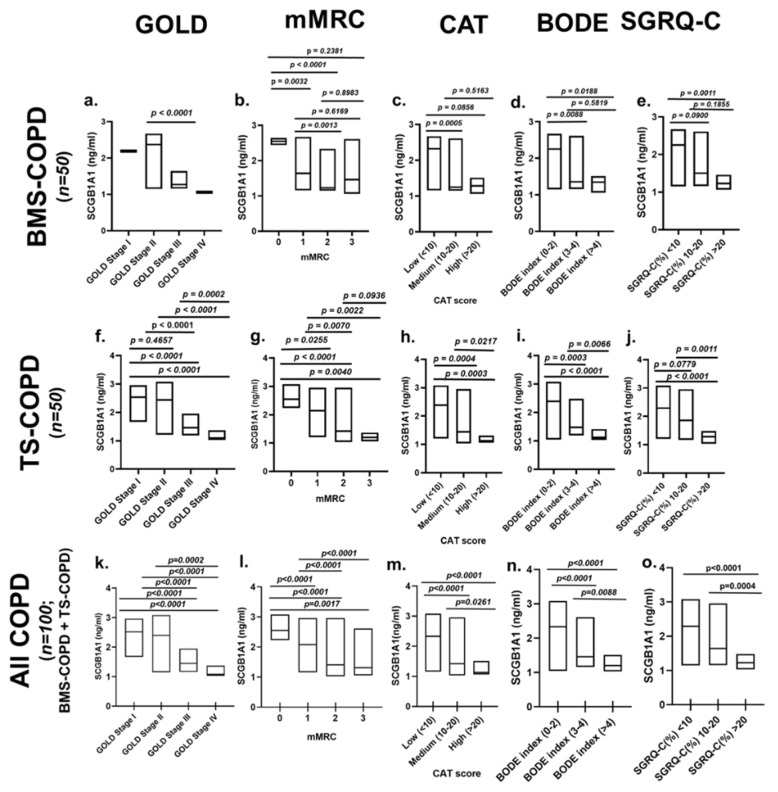
Serum concentrations of secretoglobin family 1A member 1 (SCGB1A1) among subjects with biomass smoke induced chronic obstructive pulmonary disease (BMS-COPD; *n* = 50; 39 female, 11 male; (**a**–**e**), tobacco smoke induced COPD (TS-COPD; *n* = 50, all male) (**f**–**j**) and all COPD subjects together (*n* = 100; 61 male, 39 female; (**k**–**o**)). Each subgroup is classified according to: (**a**) Severity of COPD (GOLD stages I–IV): BMS-COPD: [I: *n* = 1, II: *n* = 31, III: *n* = 17, IV: *n* = 1]; TS-COPD: [I: *n* = 10, II: *n* = 17, III: *n* = 16, IV: *n* = 7]; All COPD: [I: *n* = 11, II: *n* = 48, III: *n* = 33, IV: *n* = 8]; (**b**) Severity of breathlessness (mMRC grades 0–3): BMS-COPD: [0: *n* = 6, 1: *n* = 26, 2: *n* = 15, 3: *n* = 3]; TS-COPD: [0: *n* = 7, 1: *n* = 20, 2: *n* = 19, 3: *n* = 4]; All COPD: [0: *n* = 13, 1: *n* = 46, 2: *n* = 34, 3: *n* = 7]; (**c**) COPD assessment test (CAT) score: BMS-COPD: [low: *n* = 32; medium: *n* = 16, high: *n* = 2]; TS-COPD: [low: *n* = 26; medium: *n* = 20, high: *n* = 4]; All COPD: [low: *n* = 58; medium: *n* = 36, high: *n* = 6]; (**d**) BODE index (0–2): BMS-COPD: [0–2: *n* = 34, 3–4: *n* = 12, >4: *n* = 4]; TS-COPD: [0–2: *n* = 28, 3–4: *n* = 16, >4: *n* = 6]; All COPD: [*n* = 62, 3–4: *n* = 28, >4: *n* = 10]; (**e**) Ratings in COPD-specific version of the St. George’s Respiratory Questionnaire [SGRQ-C (%): BMS-COPD: [<10: *n* = 38; 10–20: *n* = 8, >20: *n* = 6]; TS-COPD: [<10: *n* = 27; 10–20: *n* = 14, >20: *n* = 9]; All COPD: <10: *n* = 63; 10–20: *n* = 22, >20: *n* = 15]. Data are presented as median (25th–75th percentile). Statistical analysis was performed using the non-parametric Kruskal–Wallis followed by Mann–Whitney U tests (two tailed), when appropriate. *p* < 0.05 was considered as statistically significant. BODE: body mass index (BMI, B), airflow obstruction (O), dyspnea (D), and exercise tolerance (E), CAT: COPD assessment test, GOLD: Global Initiative for Chronic Obstructive Lung Disease, mMRC: Modified Medical Research Council, SGRQ-C: COPD-specific version of the St. George’s Respiratory Questionnaire.

**Figure 4:**
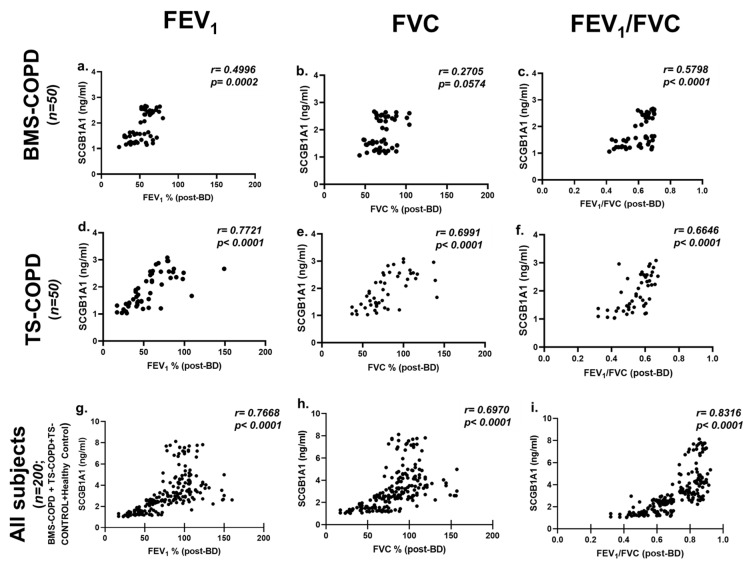
Correlation of serum concentrations of secretoglobin family 1A member 1 (SCGB1A1) with post bronchodilator (post-BD) test of forced expiratory volume in one second (FEV_1_% predicted), forced vital capacity (FVC % predicted), and FEV_1_/FVC among biomass smoke induced chronic obstructive pulmonary disease (BMS-COPD; *n* = 50; 39 female, 11 male; (**a**–**c**), tobacco smoke induced COPD (TS-COPD; *n* = 50, all male, (**d**–**f**) and all study subjects (*n* = 200; 50 TS-COPD, 50 BMS-COPD subjects, 50 TS-CONTROL, and 50 healthy control subjects) (**g**–**i**). Two tailed non-parametric Spearman’s rank test was used to analyze the correlation. *p* < 0.05 was considered as statistically significant.

Significantly decreased serum levels of SCGB1A1 in BMS-COPD subjects (total: *n* = 50; male: *n* = 11; female: *n* = 39) were detected with the severity of COPD as classified by GOLD stages (Figure 3a), mMRC grades (Figure 3b), CAT scores (Figure 3c), BODE index (Figure 3d), and SGRQ-C (%) (Figure 3e). The SCGB1A1 concentrations among the SMWD (% of expected) subgroups of BMS-COPD were not significantly different (Supplementary Table S4). Detailed analysis of the serum SCGB1A1 concentrations among BMS-COPD subjects is provided in Supplementary Table S4. A gender base stratification analysis of BMS-COPD subjects is also provided in Supplementary Table S3. A moderate positive correlation (*r* = 0.40–0.58; *p* ≤ 0.004) of serum SCGB1A1 concentrations with pre-and post-bronchodilation FEV_1_ and FEV_1_/FVC was detected in BMS-COPD subjects (Figure 4a–c, Table 2). Gender (male: *n* = 11; female: *n* = 39) stratified analysis of the correlation analysis is provided in Table 2. The same data points for TS-COPD are also provided as the disease control in Figure 3f–j, Figure 4d–f and Table 2. The difference of SCGB1A1 concentration among the SMWD (% of expected) subgroups of TS-COPD was significantly different (*p* < 0.0001; Supplementary Table S1).

While considering all the COPD subjects (*n* = 100; BMS-COPD and TS-COPD), levels of SCGB1A1 decreased with an increased severity of COPD (GOLD stages I–IV; Figure 3k) and dyspnea (mMRC; Figure 3l). Levels of SCGB1A1 also decreased with higher CAT score, BODE index, and SGRQ-C (%) index (Figure 3m–o). A gender-based stratification analysis of SCGB1A1 levels of the different sub-groups of COPD subjects according to GOLD stages, CAT score, mMRC, BODE index, SGRQ-C, and SMWD is provided in Supplementary Table S1.

Weak negative correlation of SCGB1A1 levels with age (*r* = −0.2225; *p* = 0.0015) and pack-year (*r* = −0.2020; *p* = 0.0439) was observed. However, we did not detect any correlation of SCGB1A1 levels with BMEI (*r* = 0088; *p* = 0.952). An age-based stratification analysis of the different sub-groups of study subjects is provided in Supplementary Table S2. Serum levels of SCGB1A1 were positively correlated with both pre-and post-bronchodilation FEV_1_, FVC, and FEV_1_/FVC (Figure 4g–i). Supplementary Table S3 provides the correlation analysis of pre-and post-bronchodilation FEV_1_, FVC, FEV_1_/FVC among different sub-groups of the study subjects. The difference of SCGB1A1 concentration among the SMWD (% of expected) subgroups of all COPD subjects was also significantly different (*p* < 0.0001; Supplementary Table S1).

### 3.4. SCGB1A1 Transcript and Protein Levels Following In Vitro Cigarette Smoke Condensate Exposure

**Figure 5:**
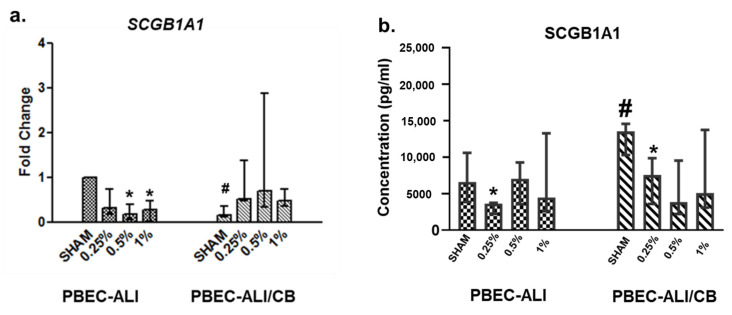
Transcript (**a**) and secreted protein levels (**b**) of secretoglobin family 1A member 1 (SCGB1A1) in the human normal-and chronic bronchitis-like (CB) bronchial mucosa models cultured at air–liquid interface (ALI) following exposure to 0.25%, 0.50% and 1% cigarette smoke condensate (CSC). Human primary bronchial epithelial cells (PBEC) from three donors (N) [three replicates/donors (*n*)] have been used to develop the bronchial mucosa models. Actin beta (*ACTB*) was used as the reference gene. Fold changes for transcript expression were calculated relative to the corresponding sham. Data are presented as median (25th–75th percentile). N = 3; *n* = 3; non-parametric statistical analysis using Friedman test followed by Wilcoxon signed rank t test as a post hoc test, when appropriate, have been performed. *p* < 0.05: *: statistically significant compared to corresponding sham; ^#^: statistically significant compared to sham exposed normal mucosa. PBEC-ALI: normal bronchial mucosa model; PBEC-ALI/CB: chronic bronchitis-like bronchial mucosa model.

None of the CSC doses used were cytotoxic for either normal (PBEC-ALI) or chronic bronchitis-like (PBEC-ALI/CB) bronchial mucosa models (Supplementary Figure S1). Significantly reduced expression of *SCGB1A1* (three to fivefold) transcripts were detected in the PBEC-ALI following exposure to 0.5% and 1% CSC (Figure 5a). Reduction of *SCGB1A1* expression following 0.25% CSC in PBEC-ALI was, however, not statistically significant (Figure 5a). Expression of *SCGB1A1* was significantly reduced (9-fold) in sham exposed PBEC-ALI/CB compared to PBEC-ALI (Figure 5a). Although, the findings in CSC exposed PBEC-ALI/CB were not statistically significant, the trends exhibit a reduced expression of *SCGB1A1* transcript compared to the corresponding sham exposed samples (Figure 5a). The SCGB1A1 protein concentration in the basal media was significantly reduced (2-fold) in case of 0.25% CSC exposed PBEC-ALI and PBEC-ALI/CB compared to the corresponding sham (Figure 5b). In case of 0.50% and 1% CSC exposure, secreted SCGB1A1 concentration in the basal media exhibited a reduced trend (statistically not significant) in both PBEC-ALI and PBEC-ALI/CB compared to corresponding sham (Figure 5b). Interestingly, the secreted protein concentration of sham exposed PBEC-ALI/CB was increased compared to sham exposed PBEC-ALI (Figure 5b). Supplementary Table S5 summarizes the findings obtained from the in vitro exposure studies.

## 4. Discussion

The main source of SCGB1A1 in serum is from the respiratory tract [19,20]. SCGB1A1-expressing cells from the bone marrow have been shown to play a role in airway epithelial regeneration [21]. SCGB1A1 is mainly produced from the club cells (non-ciliated bronchiolar cells) that are abundant in the peripheral airways contributing to the maintenance of airway integrity and repair via its anti-inflammatory, anti-oxidative, anti-toxicant, self-renewal, and differentiation properties [1,22]. Club cells also act as progenitors for the repair of bronchiolar epithelium [1,22]. Studies on murine models demonstrated the protective role of SCGB1A1 by reducing lung nuclear factor-kB (NFkB) activation following cigarette smoke exposure [23]. Cigarette smoke exposure resulted in an exacerbation of airway inflammation and alveolar loss in mice lacking *Scgb1a1* [24]. Circulating SCGB1A1 levels have also been considered as a biomarker of pollutant exposure. Nitrogen dioxide exposure levels at birth have been associated with reduced SCGB1A1 during childhood and early adulthood in the Tucson Children’s Respiratory Study [25].

To our knowledge, this is the first study on serum SCGB1A1 levels in an Indian population and to show that the SCGB1A1 levels are low in subjects with BMS-COPD. TS-COPD is used as the disease control. Consistent with other studies [20,26,27,28,29,30,31], we also found reduced serum SCGB1A1 in subjects with TS-COPD and asymptomatic smokers (TS-CONTROL) compared to healthy controls. Serum levels of SCGB1A1 of BMS-COPD and TS-COPD subjects were similar. In case of asymptomatic smokers, SCGB1A1 levels were higher than those of TS-COPD and BMS COPD subjects but lower than healthy controls. Levels of SCGB1A1 among healthy control subjects was similar to those reported earlier [29,31]. A gender specific difference of the level of SCGB1A1 among healthy controls was not observed in this study cohort consistent with the study by Rong et al. [20]. However, we detected slightly higher levels of SCGB1A1 among female BMS-COPD subjects compared to male BMS-COPD subjects. Serum SCGB1A1 levels among TS-COPD (male) and BMS-COPD (male) were not different. A strong positive correlation of serum SCGB1A1 levels was detected with lung function parameters (FEV_1_, FVC, and FEV_1_/FVC) [20,26,27,28,29,30,31] and SMWD is consistent with other studies. Decreased SCGB1A1 levels were detected with the increasing severity of COPD according to GOLD severity grading based on FEV_1_%. Further, we also detected reduced SCGB1A1 levels together with poor scores of health outcome measures relevant for COPD such as BODE index, mMRC dyspnea scores, CAT scores, and disease specific quality of life scores such as SGRQ-C. Lower levels among asymptomatic smokers compared to healthy controls but higher than that of COPD subjects further supports its protective role. Similar findings with TS-COPD subjects have been reported recently [20]. In case of the BMS-COPD sub-group, decreased serum SCGB1A1 levels have been also detected with higher GOLD stages, mMRC grade, CAT score, BODE index, and SGRQ-C (%). Taken together, reduced serum SCGB1A1 levels are a strong indicator of negative outcomes in both BMS-COPD and TS-COPD.

Reduced SCGB1A1 levels have been associated with significantly lower lung functions (FEV_1_) and airflow limitation (FEV_1_/FVC) in the general adult population after adjusting for smoking in the Spanish branch of the population-based multi-center European Community Respiratory Health Survey [29]. Several large studies [26,27,28] have confirmed the utility of SCGB1A1 as a clinical biomarker for many important health outcomes. SCGB1A1 has also been identified as an important bio-marker for FEV_1_ decline over time in the Evaluation of COPD Longitudinally to Identify Predictive Surrogate Endpoints (ECLIPSE, >3 years) [30], the Lung Health Study (>9 years) [26], and the COPDGene study (>3 years) [32]. In addition, the COPDGene study evaluated a combination of bio-markers and important health outcomes in COPD and observed that SCGB1A1 was associated with emphysema progression, airflow limitation, and mortality [32]. Guerra et al. evaluated the role of *SCGB1A1* from 3 prospective birth cohorts and 3 adult cohorts [31]. They observed in the birth cohorts that lower SCGB1A1 levels were associated with a poor lung function growth in children. In the adult cohorts, it was observed that lower SCGB1A1 levels were associated with an accelerated decline in lung function and presence of airflow limitation [31]. SCGB1A1 levels was sharply reduced in the bronchoalveolar lavage fluid of neonates that developed bronchopulmonary dysplasia [33], a risk factor for developing COPD later in life [34].

To gain insight into the local expression of SCGB1A1 transcript and protein expression following tobacco smoke exposure, we exposed physiologically relevant bronchial (normal and chronic bronchitis like) mucosa models to CSC. Chronic bronchitis is a common covariate with COPD [35]. Low circulating SCGB1A1 levels have been associated with higher risk for chronic bronchitis among smokers [36]. Consistent with the observation of reduced systemic SCGB1A1 protein concentration among COPD patients and asymptomatic smokers in this study, the normal and chronic bronchitis-like bronchial mucosa models exhibited reduction in secreted SCGB1A1 protein levels following exposure to only the lowest (0.25%) CSC concentration. Reduced transcript levels of *SCGB1A1* were detected in normal bronchial mucosa model when exposed to medium (0.5%) and high (1%) CSC concentrations. The findings are in overall supportive of the reduced expression of SCGB1A1 in the lung and its corresponding release on tobacco smoke exposure. However, we did not detect a dose gradient response SCGB1A1 transcript or protein expression following in vitro CSC exposure experiments. These observations warrant a mechanistic study focused on the regulation of SCGB1A1 following tobacco smoke and biomass smoke exposures. Exposure experiments using BMS would be more appropriate in this context as mechanistic studies revealing the cause of BMS-COPD are still lacking despite epidemiological evidence [37]. In this context, it is also important to note that in vitro exposure experiments performed in this study represent an acute exposure scenario whereas in real-life both TS-COPD and BMS-COPD result from chronic exposures. The variation between transcript and protein concentrations we observed are plausibly due to the time lag between mRNA level signal and corresponding protein release. SCGB1A1 secretion has been reported to be induced by IL13 [38,39]. Increased SCGB1A1 levels in the sham exposed chronic bronchitis-like model compared to that of normal bronchial mucosa may be explained by the addition of IL13 to induce mucous production. The reduction of SCGB1A1, the marker for airway secretory club cells may be considered indicative of impairment in the maintenance of airway integrity and repair, a common phenomenon in COPD [1]. SCGB1A1 expressing cells in the lung exert their protective function against lung injury through their progenitor, self-renewal, and differentiation properties [40]. It would be interesting to investigate how the normal and chronic bronchitis models regulate inflammatory, oxidative stress, and tissue injury response to CSC and BMS exposure. Previously, we reported an early and stronger pro-inflammatory, oxidative stress and tissue injury response in chronic bronchitis-like lung mucosa models compared to normal lung mucosa models following exposure to aerosolized carbon nanoparticles [17]. It is plausible that decreased SCGB1A1 levels observed in the bronchial mucosa models following CSC exposure is due to the loss of club cell population. Exposure studies using bronchial mucosa models to biomass smoke or biomass particles to assess SCGB1A1 transcript and protein secretion are therefore warranted.

The limitations of the study include its cross-sectional design instead of a longitudinal study, lack biomass exposed women not developing COPD sub-group, and gender imbalance (lack of female smoking subjects and limited male BMS-COPD subjects). Regarding the in vitro studies, it would have been more appropriate to expose the lung mucosal models to BMS as well in a repeated exposure setting.

## 5. Conclusions

To conclude, we detected a similar reduction of serum SCGB1A1 concentrations in subjects with tobacco smoke induced COPD and biomass smoke induced COPD. The corresponding levels of SCGB1A1 in asymptomatic smokers were higher than both TS-COPD and BMS-COPD subjects. Further, in case of healthy controls, SCGB1A1 levels were even higher than that of asymptomatic smokers. However, one needs to consider that the standard of living among healthy controls (no biomass use for cooking) was slightly higher compared to the other sub-groups even though recruited from the same region with same ethnicity. In vitro exposure studies using cigarette smoke condensate support the epidemiological findings in both normal and chronic bronchitis-like bronchial mucosa models. These observations support the protective role of SCGB1A1 against both tobacco smoke and biomass smoke induced COPD.

Consistent with these findings, levels of SCGB1A1 reduced with increased disease severity, dyspnea, poor quality of life, and higher mortality indicators. In line with previously reported studies [26,27,28,29,30,31,32,33,34], SCGB1A1 levels were positively correlated with lung function parameters in the entire study cohort, COPD subjects, and COPD male subjects. Further, the findings of the current study are also consistent with the above findings within the BMS-COPD subgroup. It is important to note that the number of BMS-COPD-male subjects (*n* = 11) was relatively small. Due to the gender imbalance in the cohort as discussed in the study limitations, an adequate number of subjects in other subgroups was lacking for such analysis. Levels of SCGB1A1 were slightly lower in case of male-BMS COPD subjects compared to female BMS-COPD subjects. This gender difference was not observed among male and female healthy controls. Levels of SCGB1A1 were also not different among TS-COPD (male) and BMS-COPD (male) subjects. Gender stratification analysis of COPD subjects for SCGB1A1 levels with disease severity, dyspnea, quality of life, and mortality indicators were in overall agreement with the findings involving the entire study cohort. Serum SCGB1A1 levels exhibited a weak negative correlation with age. Stratification analysis with age revealed, SCGB1A1 levels to be different particularly among male COPD subjects, especially among the extreme age groups.

Taken together, in our view, serum SCGB1A1 is a promising biomarker to detect subjects at risk of developing COPD, symptom/severity classified by GOLD criteria, and plausibly to assess the effect of treatment in people exposed to both tobacco smoke and biomass smoke. This can be assessed through larger well-designed independent cohorts. To understand the mechanism of SCGB1A1 regulation in the lung, mechanistic studies following repeated exposure studies using both tobacco smoke and biomass smoke in physiologically relevant lung mucosa models are warranted. Future studies focusing on these aspects will help us to more precisely understand the role of SCGB1A1 in COPD pathogenesis.

## Figures and Tables

**Table 1:** Demography and clinical characteristics of the study cohort.

	TS-COPD	TS-CONTROL	BMS-COPD	Healthy Control
Gender				
Female	0	0	39	21
Male	50	50	11	29
Total (*n*)	50	50	50	50
**Age** (years) (mean, range)	66 (51–85)	61 (40–85)	59 (40–84)	55 (40–76)
**BMEI** (median, IQR)	-(no BMS exposure during cooking)	-(no BMS exposure during cooking)	75 (60–90)	-(no BMS exposure during cooking)
**Pack-years** (median, IQR)	30 (26–33.8)	26 (22–30)	-(non-smoker)	-(non-smoker)
**Spirometry** (pre bronchodilation) (mean ± SD)				
FEV_1_% predicted	50.4 ± 23.1	99.8 ± 19.0	45.6 ± 11.7	97.0 ± 13.1
FVC% predicted	73.2 ± 26.7	102.2 ± 20.3	62.1 ± 12.2	98.9 ± 14.1
FEV_1_/FVC	0.53 ± 0.08	0.79 ± 0.05	0.60 ± 0.09	0.82 ± 0.04
**Spirometry** (post bronchodilation) (mean ± SD)				
FEV_1_% predicted	57.4 ± 25.9	103.4 ± 21.3	53 ± 13.6	100.7 ± 14.0
FVC% predicted	81.4 ± 29.5	103.3 ± 22.1	70.7 ± 13.5	99.5 ± 15.3
FEV_1_/FVC	0.55 ± 0.09	0.80 ± 0.06	0.61 ± 0.08	0.85 ± 0.05
**GOLD obstruction grading** (n)				
I	10	-	1	-
II	17	-	31	-
III	16	-	17	-
IV	7	-	1	-
**Exercise capacity** (median, IQR)				
SMWD	440 (400.5–465.6)	520.3 (461–559.6)	435 (396.3–477.5)	541.5 (510.6–556.4)
SMWD (%)	82 (75.3–90)	99.5 (96.3–104)	84 (80–90)	98 (95.3–101)
**mMRC dyspnea grading** (n)				
0	7	50	6	50
1	20	0	26	0
2	19	0	15	0
3	4	0	3	0
**CAT score** (median, IQR)	9 (6–14)	-	9 (6–12)	-
**BODE index** (median, IQR)	2 (1–4)	-	2 (1–3)	-
**SGRQ-C** (median, IQR)				
Symptom	133.2 (65.9–219.2)	-	99.55 (58.3–156.8)	-
Activity	75.7 (0–81.4)	-	75.7 (0–81.4)	-
Impact	76.1 (75.1–155.2)	-	76.1 (75.1–135.4)	-
Total score	285 (144.4–492.2)	-	231.9 (134.4–363.5)	-
SGRQ-C%	8.9 (5–15)	-	7 (4–11)	-

BMEI: Biomass exposure index, BMS: Biomass smoke, BODE: Body mass index (BMI, B), airflow obstruction (O), dyspnea (D), and exercise tolerance (E), CAT: COPD assessment test, COPD: Chronic obstructive pulmonary disease, FEV_1_: Forced expiratory volume in one second, FVC: Forced vital capacity, GOLD: Global Initiative for Chronic Obstructive Lung Disease, IQR: interquartile range (25th percentile, 75th percentile); mMRC: Modified Medical Research Council, SGRQ-C: COPD-specific version of the St. George’s Respiratory Questionnaire, SD: Standard deviation of mean; SMWD: Six-minute walk distance, TS: Tobacco smoke.

**Table 2:** Correlation of serum concentrations of secretoglobin family 1A member 1 (SCGB1A1) with pre-and post-bronchodilator (pre-BD and post-BD) test of forced expiratory volume in one second (FEV_1_% predicted), forced vital capacity (FVC % predicted), and FEV_1_/FVC among subjects with biomass smoke induced chronic obstructive pulmonary disease (BMS-COPD; male: 11, female: 39; total: *n* = 50) and tobacco smoke induced COPD (*n* = 50, all male). Two tailed non-parametric Spearman’s rank test was used to analyze the correlation. *p* < 0.05 was considered as statistically significant.

Category	n		FEV_1_		FVC		FEV_1_/FVC	
			Pre-BD	Post-BD	Pre-BD	Post-BD	Pre-BD	Post-BD
**BMS-COPD (all)**	50	*p*	**0.0015**	**0.0002**	ns (0.0585)	ns (0.0574)	**0.0038**	**<0.0001**
		*r*	0.4377	0.4996	0.2694	0.2705	0.4025	0.5798
**BMS-COPD (male)**	11	*p*	**0.0012**	ns (0.0842)	**0.0015**	ns (0.3696)	**0.0011**	**0.0116**
		*r*	0.4508	0.5484	0.4409	0.2989	0.4526	0.7432
**BMS-COPD (female)**	39	*p*	ns (0.0900)	**0.0200**	ns (0.1572)	ns (0.2521)	ns (0.2700)	**0.0041**
		*r*	0.2752	0.3712	0.2309	0.1878	0.4490	0.1811
**TS-COPD (all male)**	50	*p*	**<0.0001**	**<0.0001**	**<0.0001**	**<0.0001**	**<0.0001**	**<0.0001**
		*r*	0.7761	0.7721	0.6970	0.6991	0.6357	0.6646

**BMS:** Biomass smoke; **COPD:** Chronic obstructive pulmonary disease, **FEV_1_:** Forced expiratory volume in one second, **FVC:** Forced vital capacity, **ns:** not significant, **TS**: Tobacco smoke.

## Data Availability

All data presented in the study are available on request from the corresponding authors and are also included in the Supplementary Materials.

## Supplementary Materials

The following are available online at https://www.mdpi.com/article/10.3390/toxics9090208/s1, Figure S1: Cytotoxicity of the cigarette smoke condensate (CSC) doses on PBEC-ALI and PBEC-ALI/CB models was assessed by lactate dehydrogenase assay. Table S1: Gender based stratification analysis of serum secretoglobin family 1A member 1 (SCGB1A1) concentrations. Table S2: Age based stratification analysis of serum secretoglobin family 1A member 1 (SCGB1A1) concentrations. Table S3: Correlation of serum concentrations of secretoglobin family 1A member 1 (SCGB1A1) with pre-and post-bronchodilator (pre-BD and post-BD) test of lung function. Table S4: Serum secretoglobin family 1A member 1 (SCGB1A1) concentrations in biomass smoke induced chronic obstructive pulmonary disease (BMS-COPD) subjects according to disease severity. Table S5: Transcript and secreted protein levels of secretoglobin family 1A member 1 (SCGB1A1) in the human normal-and chronic bronchitis-like bronchial mucosa models cultured at air–liquid interface following exposure to 0.25%, 0.50% and 1% cigarette smoke condensate.
