# Supplementary material for: Circulating Secretoglobin Family 1A Member 1 (SCGB1A1) Levels as a Marker of Biomass Smoke Induced Chronic Obstructive Pulmonary Disease
Source: Toxics. 2021 Aug 31;9(9):208. doi: 10.3390/toxics9090208 (PMC8472904; doi:10.3390/toxics9090208)
Supplement: Supplementary file 1 [file toxics-09-00208-s001.zip › toxics-1307848-supplementary.pdf]

toxics

Supplementary Materials: Circulating Secretoglobin Family 1A Member 1 (SCGB1A1) Levels as a Marker of Biomass Smoke Induced Chronic Obstructive Pulmonary Disease

Vivek Vardhan Veerapaneni, Swapna Upadhyay, Tania A. Thimraj, Jayaraj Biligere Siddaiah, Chaya Sindaghatta Krishnarao, Komarla Sundararaja Lokesh, Rajesh Thimmulappa, Lena Palmberg, Koustav Ganguly and Mahesh Padukudru Anand

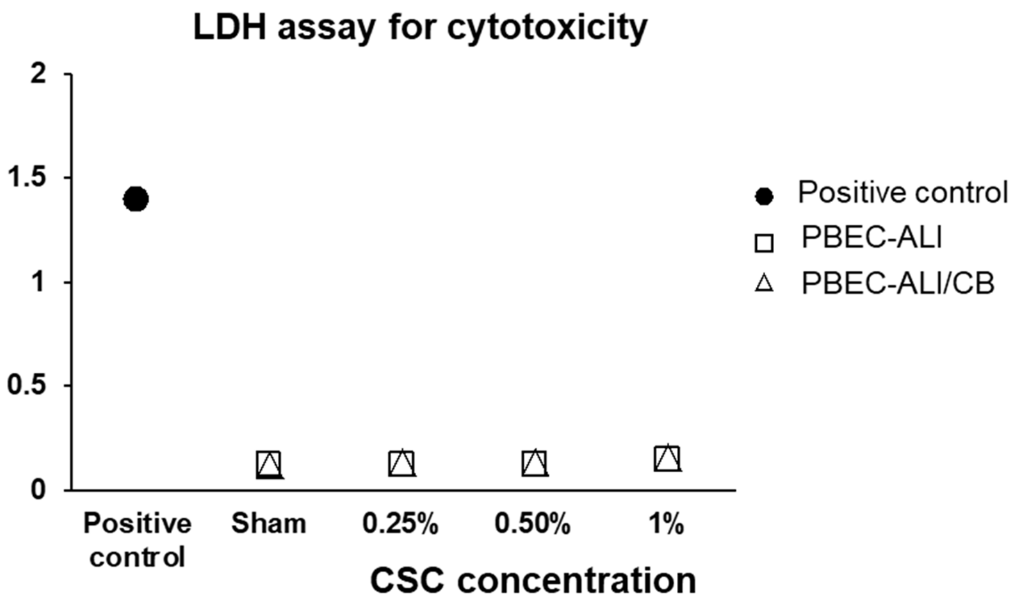

Figure S1. Cytotoxicity of the cigarette smoke condensate (CSC) doses on PBEC-ALI and PBEC-ALI/CB models was assessed by lactate dehydrogenase assay (LDH; cat# 88953, Thermo Fisher Scientific, USA) in the basal medium 24h post-exposure according to manufacturer's instruction. None of the doses were cytotoxic to either normal or chronic bronchitis-*like* bronchial mucosa model (N = 3; *n* = 3). Kit provided LDH positive control was used. PBEC-ALI: normal bronchial mucosa model; PBEC-ALI/CB: chronic bronchitis-like bronchial mucosa model.

Table S1. Gender based stratification analysis of serum secretoglobin family 1A member 1 (SCGB1A1) concentrations [median, interquartile range (IQR)] in different sub-groups of the study subjects along with corresponding statistical analysis [non-parametric Kruskal-Wallis followed by Mann Whitney U test (two tailed), when appropriate; p<0.05 was considered as significant.

**A. All study subjects**				
**Study groups**	**TS-COPD**	**TS-CONTROL**	**BMS-COPD**	**Healthy Control**
Total (*n*)	50	50	50	50
Male	*n* = 50	*n* = 50	*n* = 11	*n* = 29
Female	*n* = 0	*n* = 0	*n* = 39	*n* = 21
SCGB1A1 (ng/mL) (median, IQR)	1.80 (1.38–2.51)	3.27 (2.90–3.51)	1.61 (1.29–2.44)	5.07 (4.47–7.20)
Kruskal-Wallis test	$p < 0.0001$			
Mann-Whitney test (*p*)	**TS-COPD** vs **TS-CONTROL**		**< 0.0001**	
	**TS-COPD** vs **Healthy CONTROL**		**< 0.0001**	
	**BMS-COPD** vs **Healthy CONTROL**		**< 0.0001**	
	**BMS-COPD** vs **TS-CONTROL**		**< 0.0001**	
	BMS-COPD vs TS-COPD		ns (0.5399)	
	**TS-CONTROL** vs **Healthy control**		**< 0.0001**	
**B. Gender stratification of COPD and healthy control** (*TS-CONTROL: all 50 subjects were male and therefore not included in gender stratification analysis in this section*)				
**Study groups**	**COPD**		**Healthy Control**	
Total (*n*)	**Male**	**Female**	**Male**	**Female**
	61 (TS-COPD: *n* = 50; BMS-COPD: *n* = 11)	39 (BMS-COPD: *n* = 39)	29	21
SCGB1A1 (ng/mL) (median, IQR)	1.64 (1.31–2.44) TS-COPD: 1.80 (1.38–2.51) BMS-COPD: 1.46 (1.23–1.53)	2.02 (1.39–2.48)	4.99 (4.36–6.98)	5.82 (4.69–7.68)

Mann-Whitney test	COPD-male vs COPD-female	ns (0.9263)	ns (0.0742)	
	**BMS-COPD-male vs BMS-COPD-female**	**0.0283**		
	TS-COPD-male vs BMS-COPD-male	ns (0.0580)		
	TS-COPD-male vs BMS-COPD-female	ns (0.4630)		
**C. GOLD stage (all COPD subjects)**				
**GOLD stage**	**I**	**II**	**III**	**IV**
Total (*n* = 100)	11	48	33	08
Male (*n* = 61)	10	19	24	08
Female (*n* = 39)	01	29	09	00
SCGB1A1 (ng/ml) (median, IQR)	2.52 (2.31–2.62)	2.40 (1.62–2.55)	1.45 (1.26–1.54)	1.08 (1.06–1.21)
Kruskal-Wallis test	$p < 0.0001$			
Mann-Whitney test (*p*)	I vs II		ns (0.1801)	
	**I vs III**		**< 0.0001**	
	**I vs IV**		**< 0.0001**	
	**II vs III**		**< 0.0001**	
	**II vs IV**		**< 0.0001**	
	**III vs IV**		**0.0002**	
**D. GOLD stage (COPD male)**				
**GOLD stage**	**I**	**II**	**III**	**IV**
Total (*n* = 61)	10	19	24	08
SCGB1A1 (ng/mL) (median, IQR)	2.54 (2.34–2.65)	2.44 (2.14–2.58)	1.46 (1.28–1.54)	1.08 (1.06–1.21)
Kruskal-Wallis test	$p < 0.0001$			
Mann-Whitney test (p)	I vs II		ns (0.4218)	
	**I vs III**		**< 0.0001**	
	**I vs IV**		**< 0.0001**	

	**II vs III**		**< 0.0001**	
	**II vs IV**		**< 0.0001**	
	**III vs IV**		**0.0002**	
**E. GOLD stage (TS-COPD-male)**				
**GOLD stage**	**I**	**II**	**III**	**IV**
Total (*n* = 50)	10	17	16	07
SCGB1A1 (ng/mL) (median, IQR)	2.54 (2.34–2.65)	2.44 (2.09–2.59)	1.47 (1.38–1.66)	1.09 (1.05–1.24)
Kruskal-Wallis test	*p* = <0.0001			
Mann-Whitney test (p)	I vs II		ns (0.4657)	
	**I vs III**		**<0.0001**	
	**I vs IV**		**<0.0001**	
	**II vs III**		**0.0002**	
	**II vs IV**		**<0.0001**	
	**III vs IV**		**0.0003**	
**F. GOLD stage (BMS-COPD-male)**				
**GOLD stage**	**I**	**II**	**III**	**IV**
Total (*n* = 11)	00	02	08	01
SCGB1A1 (ng/mL) (median, IQR)	-	2.43 (2.38–2.47)	1.35 (1.23–1.50)	1.06
Kruskal-Wallis test	0.0081			
Mann-Whitney test (*p*)	**II vs III**		**0.0222**	
	III vs IV		- (only one sample in GOLD stage group IV)	
**G. GOLD stage female (BMS-COPD)**				
**GOLD stage**	**I**	**II**	**III**	**IV**
Total (*n* = 39)	01	29	09	00
SCGB1A1 (ng/mL)	2.19	2.37 (1.57–2.55)	1.27 (1.23–1.58)	-

(median, IQR)				
Kruskal-Wallis test	$p = 0.0105$			
Mann-Whitney test (p)	I vs II		- (only one sample in GOLD stage group I)	
	I vs III		- (only one sample in GOLD stage group I)	
	**II vs III**		**0.0021**	
**H. CAT Score (all COPD subjects)**				
**CAT Score**	**<10 (Low)**	**(10–20) (Medium)**	**>20 (High)**	
Total ($n = 100$)	58	36	06	
Male ($n = 61$)	27	28	06	
Female ($n = 39$)	31	08	00	
SCGB1A1 (ng/mL) (median, IQR)	2.33 (1.57–2.55)	1.42 (1.23–1.74)	1.13 (1.07–1.28)	
Kruskal-Wallis test	p<0.0001			
Mann-Whitney test (p)	**< 10 vs (10–20)**		**< 0.0001**	
	**< 10 vs >20**		**< 0.0001**	
	**(10–20) vs >20**		**0.0261**	
**I. CAT Score (COPD male)**				
**CAT Score**	**<10 (Low)**	**(10–20) (Medium)**	**>20 (High)**	
Total ($n = 61$)	27	28	06	
SCGB1A1 (ng/mL) (median, IQR)	2.44 (1.86–2.58)	1.44 (1.27–1.74)	1.13 (1.07–1.28)	
Kruskal-Wallis test	p<0.0001			
Mann-Whitney test (p)	**< 10 vs (10–20)**		**< 0.0001**	
	**< 10 vs >20**		**< 0.0001**	
	**(10–20) vs >20**		**0.0282**	
**J. CAT Score (TS-COPD-male)**				
**CAT Score**	**<10 (Low)**	**(10–20) (Medium)**	**>20 (High)**	

Total (n = 50)	26	20	04	
SCGB1A1 (ng/mL) (median, IQR)	2.39 (1.82–2.59)	1.45 (1.33–1.84)	1.13 (1.08–1.21)	
Kruskal-Wallis test	$p < 0.0001$			
Mann-Whitney test (p)	**< 10 vs (10–20)**		**0.0004**	
	**< 10 vs >20**		**0.0003**	
	**(10–20) vs >20**		**0.0217**	
**K. CAT Score (BMS-COPD-male)**				
**CAT Score**	**<10 (Low)**	**(10–20) (Medium)**	**>20 (High)**	
Total (n = 11)	01	08	02	
SCGB1A1 (ng/mL) (median, IQR)	2.52	1.35 (1.23–1.50)	1.29 (1.17–1.40)	
Kruskal-Wallis test	$p = 0.0039$			
Mann-Whitney test (p)	< 10 vs (10–20)		- [only one sample in CAT Score group <10 (low)]	
	< 10 vs >20		- [only one sample in CAT Score group <10 (low)]	
	(10–20) vs >20		ns ($p = 0.6889$)	
**L. CAT Score female (BMS-COPD)**				
**CAT Score**	**<10 (Low)**	**(10–20) (Medium)**	**>20 (High)**	
Total (n = 39)	31	08	00	
SCGB1A1 (ng/mL) (median, IQR)	2.31 (1.53–2.50)	1.25 (1.21–1.74)	-	
Mann-Whitney test (p)	**< 10 vs (10–20)**		**0.0200**	
**M. mMRC (all COPD subjects)**				
**mMRC scale**	**0**	**1**	**2**	**3**
Total (n = 100)	13	46	34	07
Male (n = 61)	07	21	27	06
Female (n = 39)	06	25	07	01

SCGB1A1 (ng/mL) (median, IQR)	2.55 (2.47–2.64)	2.08 (1.54–2.51)	1.41 (1.23–1.69)	1.31 (1.08–1.42)
Kruskal-Wallis test	$p < 0.0001$			
Mann-Whitney test (p)	**0 vs 1**		**0.0017**	
	**0 vs 2**		**< 0.0001**	
	**0 vs 3**		**0.0070**	
	**1 vs 2**		**< 0.0001**	
	**1 vs 3**		**0.0076**	
	2 vs 3		ns (0.3096)	
**N. mMRC (COPD male)**				
**mMRC scale**	**0**	**1**	**2**	**3**
Total (n = 61)	07	21	27	06
SCGB1A1 (ng/mL) (median, IQR)	2.55 (2.41–2.75)	2.21 (1.64–2.57)	1.14 (1.23–1.77)	1.20 (1.07–1.36)
Kruskal-Wallis test	$p < 0.0001$			
Mann-Whitney test (p)	0 vs 1		ns (0.0684)	
	**0 vs 2**		**< 0.0001**	
	**0 vs 3**		**0.0012**	
	**1 vs 2**		**0.0005**	
	**1 vs 3**		**0.0002**	
	2 vs 3		ns (0.0624)	
**O. mMRC (TS-COPD-male)**				
**mMRC scale**	**0**	**1**	**2**	**3**
Total (n = 50)	07	20	19	04
SCGB1A1 (ng/mL) (median, IQR)	2.61 (2.41–2.75)	2.15 (1.62–2.57)	1.42 (1.28–1.86)	1.20 (1.08–1.33)
Kruskal-Wallis test				

Mann-Whitney test (p)	**0 vs 1**		**0.0255**	
	**0 vs 2**		**0.0001**	
	**0 vs 3**		**0.0040**	
	**1 vs 2**		**0.0070**	
	**1 vs 3**		**0.0022**	
	2 vs 3		ns (0.0936)	
**P. mMRC (BMS-COPD-male)**				
**mMRC scale**	**0**	**1**	**2**	**3**
Total (n = 11)	00	01	08	02
SCGB1A1 (ng/mL) (median, IQR)	-	2.52	1.36 (1.23–1.52)	1.26 (1.16–1.36)
Kruskal-Wallis test	ns (p = 0.1576)			
**Q. mMRC female (BMS-COPD)**				
**mMRC scale**	**0**	**1**	**2**	**3**
Total (n = 39)	06	25	07	01
SCGB1A1 (ng/mL) (median, IQR)	2.55 (2.49–2.60)	1.64 (1.45–2.42)	1.23 (1.21–1.45)	2.61
Kruskal-Wallis test	$p < 0.0001$			
Mann-Whitney test (p)	**0 vs 1**		**0.0032**	
	**0 vs 2**		**0.0012**	
	0 vs 3		- (only one sample in group 3)	
	**1 vs 2**		**0.0080**	
	1 vs 3		- (only one sample in group 3)	
	2 vs 3		- (only one sample in group 3)	
**R. BODE index (all COPD subjects)**				
**BODE index**	**0–2**	**3–4**	**>4**	
Total (n = 100)	62	28	10	

Male (n = 61)	29	22	10
Female (n = 39)	33	06	00
SCGB1A1 (ng/mL) (median, IQR)	2.33 (1.57–2.55)	1.46 (1.28–1.66)	1.20 (1.07–1.41)
Kruskal-Wallis test	p<0.0001		
Mann-Whitney test (p)	**(0–2) vs (3–4)**		**< 0.0001**
	**0 vs >4**		**< 0.0001**
	**(3–4) vs >4**		**0.0088**
**S. BODE index (COPD male)**			
**BODE index**	**0–2**	**3–4**	**>4**
Total (n = 61)	29	22	10
SCGB1A1 (ng/mL) (median, IQR)	2.44 (1.95–2.59)	1.48 (1.29–1.69)	1.20 (1.07–1.41)
Kruskal-Wallis test	$p < 0.0001$		
Mann-Whitney test (p)	**(0–2) vs (3–4)**		**< 0.0001**
	**0 vs >4**		**< 0.0001**
	**(3–4) vs >4**		**< 0.0001**
**T. BODE index (TS-COPD-male)**			
**BODE index**	**0–2**	**3–4**	**>4**
Total (n = 50)	28	16	06
SCGB1A1 (ng/mL) (median, IQR)	2.40 (1.91–2.61)	1.48 (1.33–1.74)	1.13 (1.07–1.34)
Kruskal-Wallis test	$p = < 0.0001$		
Mann-Whitney test (p)	**(0–2) vs (3–4)**		**0.0003**
	**0 vs >4**		**0.0001**
	**(3–4) vs >4**		**0.0066**
**U. BODE index (BMS-COPD-male)**			

**BODE index**	**0–2**	**3–4**	**>4**
Total (*n* = 11)	01	06	04
SCGB1A1 (ng/mL) (median, IQR)	2.52	1.36 (1.23–1.53)	1.35 (1.19–1.47)
Kruskal-Wallis test	ns (*p* = 0.2381)		
**V. BODE index female (BMS-COPD)**			
**BODE index**	**0-2**	**3-4**	**>4**
Female (*n* = 39)	33	06	00
SCGB1A1 (ng/mL) (median, IQR)	2.19 (1.49–2.49)	1.36 (1.22–1.59)	-
Mann-Whitney test (*p*)	(0-2) vs (3-4)		ns (0.1052)
**W. SGRQ-C (%) (all COPD subjects)**			
**SGRQ-C (%)**	**<10**	**10–20**	**>20**
Total (*n* = 100)	63	22	15
Male (*n* = 61)	30	18	13
Female (*n* = 39)	33	04	02
SCGB1A1 (ng/mL) (median, IQR)	2.29 (1.54–2.55)	1.65 (1.36–2.15)	1.23 (1.08–1.30)
Kruskal-Wallis test	*p* < 0.0001		
Mann-Whitney test (p)	**<10 - (10–20)**		ns (0.0599)
	**<10 vs >20**		**< 0.0001**
	**<(10–20) vs >20**		**0.0004**
**X. SGRQ-C (%) (COPD male)**			
**SGRQ-C (%)**	**<10**	**10–20**	**>20**
Male (*n* = 61)	30	18	13
SCGB1A1 (ng/mL) (median, IQR)	2.31 (1.57–2.57)	1.61 (1.36–2.13)	1.23 (1.06–1.31)

Kruskal-Wallis test	$p < 0.0001$		
Mann-Whitney test (*p*)	**<10 - (10–20)**		**0.0261**
	**<10 vs >20**		**< 0.0001**
	**<(10–20) vs >20**		**0.0008**
**Y. SGRQ-C (%) (TS-COPD-male)**			
**SGRQ-C (%)**	**<10**	**10–20**	**>20**
Male ($n = 50$)	27	14	09
SCGB1A1 (ng/mL) (median, IQR)	2.29 (1.60–2.57)	1.86 (1.43–2.32)	1.28 (1.06–1.31)
Kruskal-Wallis test	0.0002		
Mann-Whitney test (*p*)	<10 - (10–20)		ns (0.1779)
	**<10 vs >20**		**<0.0001**
	**<(10–20) vs >20**		**0.0011**
**Z. SGRQ-C (%) (BMS-COPD-male)**			
**SGRQ-C (%)**	**<10**	**10–20**	**>20**
Male ($n = 11$)	03	04	04
SCGB1A1 (ng/mL) (median, IQR)	2.33 (1.94–2.43)	1.36 (1.21–1.50)	1.23 (1.19–1.29)
Kruskal-Wallis test	0.0194		
Mann-Whitney test (*p*)	<10 - (10–20)		ns (0.0571)
	<10 vs >20		ns (0.0571)
	<(10–20) vs >20		ns (0.4857)
**AA. SGRQ-C (%) female (BMS-COPD)**			
**SGRQ-C (%)**	**<10**	**10–20**	**>20**
Female ($n = 39$)	33	04	02
SCGB1A1 (ng/mL) (median, IQR)	2.19 (1.45–2.49)	1.80 (1.49–2.17)	1.25 (1.24–1.25)

Kruskal-Wallis test	ns (0.1995) did not proceed with Mann-Whitney test	
**AB. SMWD (% of predicted) (all COPD subjects)**		
**SMWD**	**50–79**	**≥80**
Total (*n* = 100)	31	69
Male (*n* = 61)	26	35
Female (*n* = 39)	05	34
SCGB1A1 (ng/mL) (median, IQR)	1.45 (1.21–1.68)	2.21 (1.47–2.55)
Mann-Whitney test (*p*)	**50–79 vs ≥80**	**<0.0001**
**AC. SMWD (% of predicted) (COPD male)**		
**SMWD**	**50–79**	**≥80**
Male (*n* = 61)	26	35
SCGB1A1 (ng/mL) (median, IQR)	1.36 (1.18–1.61)	2.33 (1.54–2.57)
Mann-Whitney test (*p*)	**50–79 vs ≥80**	**<0.0001**
**AD. SMWD (% of predicted) (TS-COPD-male)**		
**SMWD**	**50–79**	**≥80**
Male (*n* = 50)	20	30
SCGB1A1 (ng/mL) (median, IQR)	1.36 (1.19–1.74)	2.35 (1.58–2.59)
Mann-Whitney test (*p*)	**50–79 vs ≥80**	**<0.0001**
**AE. SMWD (% of predicted) (BMS-COPD-male)**		
**SMWD**	**50–79**	**≥80**
Male (*n* = 11)	6	5
SCGB1A1 (ng/mL) (median, IQR)	1.35 (1.18–1.48)	1.54 (1.23–2.33)
Mann-Whitney test (*p*)	50–79 vs ≥80	ns (0.1797)

**AF. SMWD (% of predicted) female (BMS-COPD)**		
**SMWD**	**50–79**	**≥80**
Female (*n* = 39)	05	34
SCGB1A1 (ng/mL) (median, IQR)	1.64 (1.45–2.37)	2.05 (1.36–2.49)
Mann-Whitney test (*p*)	50–79 vs ≥80	ns (0.8148)

BMEI: Biomass exposure index, BMS: Biomass smoke, BODE: Body mass index (BMI, B), airflow obstruction (O), dyspnea (D), and exercise tolerance (E), CAT: COPD assessment test, COPD: Chronic obstructive pulmonary disease, FEV_1: Forced expiratory volume in one second, FVC: Forced vital capacity, GOLD: Global Initiative for Chronic Obstructive Lung Disease, IQR: interquartile range (25th percentile, 75th percentile); mMRC: Modified Medical Research Council, SGRQ-C: COPD-specific version of the St. George's Respiratory Questionnaire, ns: not significant, SMWD: Six-minute walk distance, TS: Tobacco smoke

Table S2. Age based stratification analysis of serum secretoglobin family 1A member 1 (SCGB1A1) concentrations [median, interquartile range (IQR)] in different sub-groups of the study subjects along with corresponding statistical analysis [non-parametric Kruskal-Wallis followed by Mann Whitney U test (two tailed), when appropriate; p<0.05 was considered as significant].

**A. Age (all subjects)**				
**Age groups**	**40–49y**	**50–59y**	**60–69y**	**≥70y**
Total (*n* = 200)	29	65	70	36
Male (*n* =140)	*n* = 13	*n* = 42	*n* = 53	*n* = 32
Female (*n* = 60)	*n* = 16	*n* = 23	*n* = 17	n = 04
SCGB1A1 (ng/mL) (median, IQR)	3.51 (2.65–6.66)	3.03 (1.46–4.46)	2.55 (1.59–3.42)	2.58 (1.78–3.10)
Kruskal-Wallis test	*p* = 0.0041			
Mann-Whitney test (*p*)	(40–49y) vs (50–59y)		ns (0.0512)	
	**(40–49y) vs (60–69y)**		**0.0004**	
	**(40–49y) vs ≥70y**		**0.0008**	
	(50–59y) vs (60–69y)		ns (0.1965)	
	(50–59y) vs ≥70y		ns (0.1793)	
	(60–69y) vs ≥70y		ns (0.9854)	
**B. Age (all male subjects)**				
**Age groups**	**40–49y**	**50–59y**	**60–69y**	**≥70y**
Male (*n* =140)	*n* = 13	*n* = 42	*n* = 53	*n* = 32
SCGB1A1 (ng/mL) (median, IQR)	3.36 (3.12–4.14)	3.27 (1.52–4.16)	2.61 (1.71–3.51)	2.63 (1.87–3.19)
Kruskal-Wallis test	*p* = 0.0209			
Mann-Whitney test (*p*)	(40–49y) vs (50–59y)		ns (0.1985)	
	**(40–49y) vs (60–69y)**		**0.0030**	
	**(40–49y) vs ≥70y**		**0.0016**	
	(50–59y) vs (60–69y)		ns (0.2017)	
	(50–59y) vs ≥70y		ns (0.1593)	
	(60–69y) vs ≥70y		ns (0.9586)	
**C. Age (all female subjects)**				

**Age groups**	**40–49y**	**50–59y**	**60–69y**	**≥70y**
Female (*n* = 60)	*n* = 16	*n* = 23	*n* = 17	*n* = 04
SCGB1A1 (ng/mL) (median, IQR)	4.25 (2.28–7.33)	2.44 (1.44–4.95)	2.47 (1.57–2.62)	1.85 (1.56–2.21)
Kruskal-Wallis test	ns ($p = 0.2156$) did not proceed with Mann-Whitney test			
**D. Age (all COPD subjects)**				
**Age groups**	**40–49y**	**50–59y**	**60–69y**	**≥70y**
Total (*n* = 100)	07	29	41	23
Male (*n* = 61)	00	15	27	19
Female (*n* = 39)	07	14	14	04
SCGB1A1 (ng/mL) (median, IQR)	2.02 (1.43–2.46)	1.43 (1.23–2.29)	1.77 (1.47–2.49)	2.07 (1.49–2.58)
Kruskal-Wallis test	ns ($p = 0.05$) did not proceed with Mann-Whitney test			
**E. Age (all male COPD subjects)**				
**Age groups**	**40–49y**	**50–59y**	**60–69y**	**≥70y**
Male (*n* = 61)	00	15	27	19
SCGB1A1 (ng/mL) (median, IQR)	-	1.31 (1.22–1.56)	1.71 (1.42–2.40)	2.21 (1.49–2.58)
Kruskal-Wallis test	p = 0.0256			
Mann-Whitney test (*p*)	(50–59y) vs (60–69y)		ns ($p = 0.0537$)	
	**(50–59y) vs ≥70y**		$\mathbf{p = 0.0086}$	
	(60–69y) vs ≥70y		ns ($p = 0.2603$)	
**F. Age (all female COPD subjects)**				
**Age groups**	**40–49y**	**50–59y**	**60–69y**	**≥70y**
Female (*n* = 39)	07	14	14	04
SCGB1A1 (ng/mL) (median, IQR)	2.02 (1.43–2.46)	1.55 (1.24–2.41)	2.38 (1.57–2.54)	1.85 (1.56–2.21)
Kruskal-Wallis test	ns ($p = 0.5563$) did not proceed with Mann-Whitney test			
**G. Age (all male TS-Control and Healthy Control subjects)**				
**Age groups**	**40–49y**	**50–59y**	**60–69y**	**≥70y**
Total (*n* = 79)	13	27	26	13

SCGB1A1 (ng/mL) (median, IQR)	3.36 (3.12–4.14)	3.77 (3.29–4.50)	3.54 (2.74–4.44)	3.38 (2.97–4.04)
Kruskal-Wallis test	ns (*p* = 0.4342) did not proceed with Mann-Whitney test			
**H. Age (all male TS Control subjects)**				
**Age groups**	**40–49y**	**50–59y**	**60–69y**	**≥70y**
Total (*n* = 50)	08	15	17	10
SCGB1A1 (ng/mL) (median, IQR)	2.61 (2.54–2.63)	2.96 (2.87–3.06)	3.31 (3.29–3.47)	3.89 (3.76–4.02)
Kruskal-Wallis test	ns (*p* = 0.4021) did not proceed with Mann-Whitney test			
**I. Age (all male Healthy Control subjects)**				
**Age groups**	**40–49y**	**50–59y**	**60–69y**	**≥70y**
Total (*n* = 29)	05	12	09	03
SCGB1A1 (ng/mL) (median, IQR)	5.43 (4.14–6.73)	4.76 (4.33–7.04)	4.99 (4.49–6.83)	4.79 (4.58–5.89)
Kruskal-Wallis test	ns (*p* = 0.9912) did not proceed with Mann-Whitney test			
**J. Age (all female Healthy Control subjects)**				
**Age groups**	**40–49y**	**50–59y**	**60–69y**	**≥70y**
Total (*n* = 21)	09	09	03	00
SCGB1A1 (ng/mL) (median, IQR)	7.21 (4.59–7.75)	5.82 (4.91–7.26)	4.89 (4.59–5.02)	-
Kruskal-Wallis test	ns (*p* = 0.5883) did not proceed with Mann-Whitney test			

BMEI: Biomass exposure index, BMS: Biomass smoke, BODE: Body mass index (BMI, B), airflow obstruction (O), dyspnea (D), and exercise tolerance (E), CAT: COPD assessment test, COPD: Chronic obstructive pulmonary disease, FEV_1: Forced expiratory volume in one second, FVC: Forced vital capacity, GOLD: Global Initiative for Chronic Obstructive Lung Disease, IQR: interquartile range (25th percentile, 75th percentile); mMRC: Modified Medical Research Council, SGRQ-C: COPD-specific version of the St. George's Respiratory Questionnaire, ns: not significant, SMWD: Six-minute walk distance, TS: Tobacco smoke.

Table S3. Correlation of serum concentrations of secretoglobin family 1A member 1 (SCGB1A1) with pre- and post- bronchodilator (pre-BD and post-BD) test of forced expiratory volume in one second (FEV_1% predicted), forced vital capacity (FVC % predicted), and FEV_1/FVC among the different sub-groups of the study subjects Two tailed non-parametric Spearman's rank test was used to analyze the correlation. $p < 0.05$ was considered as statistically significant.

Category	n	Statistic	FEV_1		FVC		FEV_1/FVC	
			Pre-BD	Post-BD	Pre-BD	Post-BD	Pre-BD	Post-BD
All subjects	200	*p*	**<0.0001**	**<0.0001**	**<0.0001**	**<0.0001**	**<0.0001**	**<0.0001**
		r	0.7815	0.7688	0.6922	0.6970	0.7942	0.8316
COPD	100	*p*	**<0.0001**	**<0.0001**	**<0.0001**	**<0.0001**	**<0.0001**	**<0.0001**
		r	0.6470	0.6768	0.5700	0.5335	0.4472	0.5482
COPD-male	61	*p*	**<0.0001**	**<0.0001**	**<0.0001**	**<0.0001**	**<0.0001**	**<0.0001**
		r	0.8616	0.8359	0.7429	0.6726	0.8574	0.7103
COPD-female (BMS-COPD)	39	*p*	ns (0.0900)	**0.0200**	ns (0.1572)	ns (0.2521)	0.2700	**0.0041**
		r	0.2752	0.3712	0.2309	0.1878	0.4490	0.1811
TS -CONTROL + Healthy Control	100	*p*	ns (0.4713)	ns (0.6571)	ns (0.8555)	ns (0.7121)	ns (0.1928)	**0.0030**
		r	−0.0729	−0.0449	−0.0185	−0.0374	0.1313	0.2935
TS -CONTROL (male)	50	*p*	ns (0.8552)	ns (0.9077)	ns (0.0638)	ns (0.5899)	**0.0187**	ns (0.1613)
		r	−0.0265	0.0168	0.0752	0.0781	−0.3314	−0.2012
Healthy Control (29 male, 21 female)	50	*p*	ns (0.5908)	ns (0.3261)	ns (0.3119)	ns (0.2496)	ns (0.8982)	ns (0.8422)
		r	−0.0779	−0.1418	−0.1459	−0.1659	−0.0186	−0.0289

Healthy Control-male	29	*p*	ns (0.3581)	ns (0.0998)	ns (0.0698)	ns (0.0876)	ns (0.9363)	ns (0.8899)
		r	−0.1771	−0.3117	−0.3415	−0.3228	−0.0155	−0.0269
Healthy Control-female	21	*p*	ns (0.7301)	ns (0.6241)	ns (0.5719)	ns (0.7428)	ns (0.9019)	ns (0.7892)
		r	0.0801	0.1135	0.1308	0.0762	−0.0287	0.0621

BMS: Biomass smoke; COPD: Chronic obstructive pulmonary disease, FEV_1: Forced expiratory volume in one second, FVC: Forced vital capacity, TS: Tobacco smoke.

Table S4. Serum secretoglobin family 1A member 1 (SCGB1A1) concentrations [median, interquartile range (IQR)] in biomass smoke induced COPD subjects (n=50; male: 11, female 39) classified according to GOLD stage, CAT score, mMRC grade, SGRQ-C% and SMWD along with corresponding statistical analysis [non-parametric Kruskal-Wallis followed by Mann Whitney U test (two tailed), when appropriate; p<0.05 was considered as significant].

**A. GOLD stage (all BMS-COPD subjects)**				
**GOLD stage**	**I**	**II**	**III**	**IV**
Total (*n* = 50)	01	31	17	01
Male (*n* = 11)	00	02	08	01
Female (*n* = 39)	01	29	09	00
SCGB1A1 (ng/mL) (median, IQR)	2.19	2.37 (1.57–2.54)	1.27 (1.23–1.51)	1.06
Kruskal-Wallis test	*p* = 0003			
Mann-Whitney test (*p*)	I vs II		- (only one sample in GOLD stage group I)	
	I vs III		- (only one sample in GOLD stage group I)	
	I vs IV		- (only one sample in GOLD stage groups I and IV)	
	**II vs III**		**<0.0001**	
	II vs IV		- (only one sample in GOLD stage group IV)	
	III vs IV		- (only one sample in GOLD stage group IV)	
**B. CAT Score (all BMS-COPD subjects)**				
**CAT Score**	**<10 (Low)**	**(10–20) (Medium)**	**>20 (High)**	
Total (*n* = 50)	32	16	02	
Male (*n* = 11)	01	08	02	

Female (n = 39)	31	08	00	
SCGB1A1 (ng/mL) (median, IQR)	2.32 (1.55–2.51)	1.25 (1.23–1.57)	1.29 (1.17–1.40)	
Kruskal-Wallis test	p = 0015			
Mann-Whitney test (p)	**< 10 vs (10–20)**		**0.0005**	
	< 10 vs >20		ns (0.0856)	
	(10–20) vs >20		ns (0.5163)	
**C. mMRC (all BMS-COPD subjects)**				
**mMRC scale**	**0**	**1**	**2**	**3**
Total (n = 50)	06	26	15	03
Male (n = 11)	00	01	08	02
Female (n = 39)	06	25	07	01
SCGB1A1 (ng/mL) (median, IQR)	2.55 (2.49–2.60)	1.64 (1.45–2.42)	1.23 (1.22–1.53)	1.46 (1.26–2.04)
Kruskal-Wallis test	p = 0004			
Mann-Whitney test (p)	**0 vs 1**		**0.0032**	
	**0 vs 2**		**< 0.0001**	
	0 vs 3		ns (0.2381)	
	**1 vs 2**		**0.0013**	
	1 vs 3		ns (0.6169)	
	2 vs 3		ns (0.8983)	
**D. BODE index (all BMS-COPD subjects)**				
**BODE index**	**0–2**	**3–4**	**>4**	
Total (n = 50)	34	12	04	
Male (n = 11)	01	06	04	
Female (n = 39)	33	06	00	
SCGB1A1 (ng/mL) (median, IQR)	2.25 (1.51–2.51)	1.36 (1.23–1.57)	1.35 (1.19–1.47)	
Kruskal-Wallis test	p = 0060			
Mann-Whitney test (p)	**(0–2) vs (3–4)**		**0.0088**	

	**0 vs >4**		**0.0188**
	(3–4) vs >4		ns (0.5819)
**E. SGRQ-C (%) (all BMS-COPD subjects)**			
**SGRQ-C (%)**	**<10**	**10–20**	**>20**
Total (*n* = 50)	38	08	06
Male (*n* = 11)	03	04	04
Female (*n* = 39)	33	04	02
SCGB1A1 (ng/mL) (median, IQR)	2.25 (1.48–2.50)	1.50 (1.23–1.69)	1.23 (1.23–1.25)
Kruskal-Wallis test	$p = 0.0043$		
Mann-Whitney test (*p*)	**<10 - (10-20)**		ns (0.0900)
	**<10 vs >20**		**0.0011**
	**<(10-20) vs >20**		ns (0.1855)
**F. SMWD (% of expected) (all BMS-COPD subjects)**			
**SMWD**	**50–79**	**≥80**	
Total (*n* = 50)	11	39	
Male (*n* = 11)	06	05	
Female (*n* = 39)	05	34	
SCGB1A1 (ng/mL) (median, IQR)	1.46 (1.23–1.58)	2.02 (1.34–2.48)	
Mann-Whitney test (*p*)	**(50–79) vs ≥80**	ns (0.0756)	

BMS: Biomass smoke, BODE: Body mass index (BMI, B), airflow obstruction (O), dyspnea (D), and exercise tolerance (E), CAT: COPD assessment test, COPD: Chronic obstructive pulmonary disease, FEV_1: Forced expiratory volume in one second, FVC: Forced vital capacity, GOLD: Global Initiative for Chronic Obstructive Lung Disease, IQR: interquartile range (25th percentile, 75th percentile); mMRC: Modified Medical Research Council, SGRQ-C: COPD-specific version of the St. George's Respiratory Questionnaire, ns: not significant, SMWD: Six-minute walk distance.

Table S5. Transcript and secreted protein levels of secretoglobin family 1A member 1 (SCGB1A1) in the human normal- and chronic bronchitis-like (CB) bronchial mucosa models cultured at air-liquid interface (ALI) following exposure to 0.25%, 0.50% and 1% cigarette smoke condensate (CSC). Three donors (N = 3) and three replicates/donors (*n* = 3) have been used.

**A. *SCGB1A1* transcript expression in PBEC-ALI** (N = 3; *n* = 3)				
	**0.25% CSC**	**0.5% CSC**	**1% CSC**	**SHAM PBEC-ALI/CB**
**Fold change** (median, IQR) **compared to SHAM-PBEC-ALI**	0.31 (0.18–0.74)	0.18 (0.05–0.39)	0.28 (0.02–0.47)	0.16 (0.11–0.35)
**Friedman's test**	***p* = 0.0155**			
**Wilcoxon signed rank test (*p*)**	SHAM-PBEC-ALI vs 0.25%			**0.0499**
	SHAM-PBEC-ALI vs 0.5%			**0.0117**
	SHAM-PBEC-ALI vs 1%			**0.0251**
	SHAM-PBEC-ALI vs PBEC-ALI/CB			**0.0284**

**B. *SCGB1A1* transcript expression in PBEC-ALI/CB** (N = 3; *n* = 3)			
	**0.25% CSC**	**0.5% CSC**	**1% CSC**
**Fold change** (median, IQR) **compared to SHAM-PBEC-ALI/CB**	0.52 (0.47–1.38)	0.69 (0.33–2.87)	0.47 (0.35–0.74)
**Friedman's test**	ns (*p* = 0.2688); did not proceed with Wilcoxon signed rank test		

**C. Secreted SCGB1A1 protein concentration in PBEC-ALI** (N = 3; *n* = 3)

	**SHAM-PBEC-ALI**	**0.25% CSC**	**0.5% CSC**	**1% CSC**	**SHAM-PBEC-ALI/CB**
**SCGB1A1 concentration (median, IQR pg/ml)**	6616 (4615–10213)	3626 (3344–3714)	7050 (4032–9368)	4485 (2958–7834)	13539 (7801–14259)
**Friedman's test**	***p* = 0.0492**				
**Wilcoxon signed rank test** (*p*)	SHAM-PBEC-ALI vs 0.25%				***p* = 0.0421**
	SHAM-PBEC-ALI vs 0.5%				ns (*p* = 0.9340)
	SHAM-PBEC-ALI vs 1%				ns (*p* = 0.9842)
	SHAM-PBEC-ALI vs PBEC-ALI/CB				***p* = 0.0314**
**D. Secreted SCGB1A1 protein concentration in PBEC-ALI/CB** (N = 3; *n* = 3)					
		**SHAM-PBEC-ALI/CB**	**0.25% CSC**	**0.5% CSC**	**1% CSC**
**SCGB1A1 concentration (median, IQR pg/ml)**		13539 (7801–14259)	7619 (4018–9022)	3732 (1750–9441)	5072 (3468–13539)
**Friedman's test**	***p* = 0.0239**				
**Wilcoxon signed rank test** (*p*)		SHAM-PBEC-ALI/CB vs 0.25%			***p* = 0.0362**
		SHAM-PBEC-ALI/CB vs 0.5%			ns (*p* = 0.2218)
		SHAM-PBEC-ALI/CB vs 1%			ns (*p* = 0.2904)

IQR: interquartile range, ns: not significant, PBEC: human primary bronchial epithelial cells, PBEC-ALI/CB: chronic bronchitis-like bronchial mucosa model, PBEC-ALI: normal bronchial mucosa model.
